# The methylenetetrahydrofolate reductase c.c.677 C>T and c.c.1298 A>C polymorphisms in reproductive failures: Experience from an RSA and RIF study on a Polish population

**DOI:** 10.1371/journal.pone.0186022

**Published:** 2017-10-26

**Authors:** Izabela Nowak, Aleksandra Bylińska, Karolina Wilczyńska, Andrzej Wiśniewski, Andrzej Malinowski, Jacek R. Wilczyński, Paweł Radwan, Michał Radwan, Ewa Barcz, Rafał Płoski, Hanna Motak-Pochrzęst, Małgorzata Banasik, Maciej Sobczyński, Piotr Kuśnierczyk

**Affiliations:** 1 Department of Clinical Immunology, Laboratory of Immunogenetics and Tissue Immunology, Hirszfeld Institute of Immunology and Experimental Therapy, Polish Academy of Sciences, Wrocław, Poland; 2 Department of Surgical, Endoscopic and Oncologic Gynecology, Polish Mothers’ Memorial Hospital–Research Institute, Łódź, Poland; 3 Department of Gynecology and Gynecologic Oncology, Polish Mothers’ Memorial Hospital–Research Institute, Łódź, Poland; 4 Department of Reproductive Medicine, Gameta Hospital, Rzgów, Poland; 5 Biogeno–Regional Science-Technology Center, Podzamcze, Poland; 6 First Chair and Clinic of Obstetrics and Gynecology, Medical University of Warsaw, Warszawa, Poland; 7 Department of Medical Genetics, Center of Biostructure Research, Medical University of Warsaw, Warszawa, Poland; 8 Faculty of Physical Education and Physiotherapy, Opole University of Technology, Opole, Poland; 9 Obstetric Gynecological Department, District Hospital Strzelce Opolskie, Strzelce Opolskie, Poland; 10 APC–Medical Analyses, Łódź, Poland; 11 Department of Genomics, Faculty of Biotechnology, University of Wrocław, Wrocław, Poland; Universite Clermont Auvergne, FRANCE

## Abstract

Almost 1600 individuals from the Polish population were recruited to this study. Among them 319 were fertile couples, 289 were recurrent spontaneous abortion (RSA) couples, and 131 were in the group of recurrent implantation failure (RIF) following in vitro fertilization. The aim of this study was to evaluate the *MTHFR* c.c.677 C>T and c.c.1298 A>C polymorphisms’ association with RSA and RIF. We used PCR-RFLP with *Hinf*I (677 C>T) and *Mbo*II (1298 A>C) digestion. We observed a protective effect of the female AC genotype (OR = 0.64, *p* = 0.01) and the C allele (AC+CC genotypes; OR = 0.65, *p* = 0.009) against RSA. Moreover, 1298 AA/677 CT women were more frequent in RSA (31.14%) and RIF (25.20%) groups in comparison to fertile women (22.88%), although this difference was significant only in the case of RSA (*p* = 0.022, OR = 1.52). Male combined genotype analysis revealed no association with reproductive failure of their partners. Nevertheless, the female/male combination AA/AC of the 1298 polymorphism was more frequent in RSA couples (*p* = 0.049, OR = 1.49). However, the significant results became insignificant after Bonferroni correction. In addition, analysis of haplotypes showed significantly higher frequency of the C/C haplotype (1298 C/677 C) in the female control group than in the female RSA group (*p* = 0.03, OR = 0.77). Moreover, the association between elevated homocysteine (Hcy) level in plasma of RSA and RIF women and *MTHFR* polymorphisms was investigated but did not reveal significant differences. In conclusion, for clinical practice, it is better to check the homocysteine level in plasma and, if the Hcy level is increased, to recommend patients to take folic acid supplements rather than undergo screening of *MTHFR* for 1298 A>C and 677 C>T polymorphisms.

## Introduction

Nowadays, recurrent spontaneous abortion (RSA) and recurrent implantation failure after *in vitro* fertilization (RIF) are implicated as the most frustrating clinical conditions for both couples who have problems with conceiving and maintaining pregnancy and for doctors who cannot help patients, especially in RIF cases [1]. Natural conception in the female body remains to some extent an enigmatic phenomenon, whereas *in vitro* fertilization (IVF) with subsequent embryo transfer provides clinicians some information about when the embryo was transferred and whether implantation has actually occurred. Hence, recurrent implantation failure should become a clinically identifiable phenomenon. Unfortunately, clinicians are still observing increasing numbers of RSA and RIF cases and they are implementing expanded diagnostics in the quest for the cause of this situation as soon as a woman experiences 2 consecutive miscarriages or cannot become pregnant after a repeated IVF. The incidence of RSA in women of reproductive age has been reported to be 1–2%, and about 60% of these were idiopathic [2,3]. The RIF prevalence rate in women following IVF treatment is approximately 10% [4].

Many factors contribute to these reproductive failures, including the woman's age, oocyte and sperm quality, parental chromosomal anomalies, genetic or metabolic abnormalities of the embryo, and poor uterine receptivity. What is more, immunological disturbances in the implantation site, endometriosis, uterine fibroids, hydrosalpinx, and endometrial polyps could negatively affect the implantation rate [1,4–6]. Both inherited and acquired thrombophilic factors are also believed to impair the reproductive outcome by thrombosis of maternal vessels causing a diminished perfusion of the intervillous space, and therefore leading to defect of implantation and placentation [7–9].

One of the key enzymes which could affect the thrombotic status and folate metabolism in humans is methylenetetrahydrofolate reductase (MTHFR). It catalyzes formation of 5-methyltetrahydrofolate, which is involved in the methylation of homocysteine (Hcy) to methionine. The C to T substitution in the c.c.677 position in the *MTHFR* gene (rs1801133, OMIM accession number 607093.0003) leads to an amino acid change from alanine to valine within the N-terminal catalytic domain of the enzyme and subsequent reduced enzymatic activity of MTHFR. A second common variant, which is also implicated in the reduction of MTHFR enzyme activity, is the c.c.1298 A>C polymorphism (rs1801131, OMIM accession number 607093.0004). It leads to substitution of glutamate to alanine in the C-terminal regulatory domain of the enzyme. The *MTHFR* 677 TT genotype results in 30% enzyme activity *in vitro* compared with the CC wild-type, whereas the *MTHFR* 1298 CC genotype has been found to result in 60% enzyme activity *in vitro* compared to the AA wild-type. No individuals were found homozygous for both mutations [10–12]. In turn, decreased enzymatic activity of MTHFR entails increased concentrations of Hcy in body fluids, especially when the folate status is low. Elevated plasma Hcy levels are associated with increased odds of venous and arterial thrombosis. An excess of homocysteine can affect the developing gametes and embryo by DNA damage and improper methylation [13]. Both polymorphisms have been associated with DNA hypomethylation [14].

Whereas *MTHFR* polymorphisms have been widely investigated in RSA case-control and meta-analysis studies [15–17], the association of RIF with *MTHFR* polymorphisms has been evaluated in only a few studies [7,18–20]. The results and conclusions were contradictory, even between meta-analysis studies [8,15,21]. Most of the studies have referred only to women, although testing male partners might be important too, owing to the fact that *MTHFR* polymorphisms in 677 C>T and 1298 A>C were reported to be associated with worse parameters of semen and male infertility [13,22,23]. Therefore, we decided to include men in our research as well.

The aim of this study was to evaluate the association between *MTHFR* 677 C>T and 1298 A>C polymorphisms and the reproductive outcome in RSA and RIF in the Polish population. This is the first such large study involving a homogeneous population in which women and their partners from control, RSA, and RIF groups of reproductive age were compared. Likewise, we analyzed the role of *MTHFR* polymorphism on the homocysteine level detected in plasma of RSA and RIF female patients.

## Materials and methods

### Study design

Almost 1600 individuals from the Polish population were originally included in this study. All spontaneous miscarriage patients (and their partners) were recruited from the Department of Surgical, Endoscopic and Oncologic Gynecology and the Department of Gynecology and Gynecologic Oncology, Polish Mothers’ Memorial Hospital–Research Institute, and the Clinic of Obstetrics and Gynecology, Medical University of Warsaw. We tested 289 couples, who suffered from two to eight consecutive spontaneous miscarriages (RSA). The inclusion of RSA couples in the study took place according to recommendations of the Practice Committee of the American Society for Reproductive Medicine, 2013 [24]. Couples, free from chromosomal aberrations (with euploid karyotypes of parents), uterine anomalies, hormonal disturbances, and infections with *Toxoplasma*, *Chlamydia*, *Listeria*, and *Brucella*, were qualified. This group consisted of 205 women who experienced 3 or more first trimester spontaneous abortion incidents with the same partner (mean age 32.82 years ± 4.02; age range 24–46) and 84 women with 2 miscarriages (mean age 32.82 years ± 4.03; age range 25–41). Nearly all women were primary aborters and miscarriages recurred at the same gestational age up to 12 weeks of gestation (98%).

One hundred thirty-one recurrent implantation failure couples were recruited from the Department of Reproductive Medicine, Gameta Hospital and Polish Mothers’ Memorial Hospital–Research Institute. RIF was defined as the failure to achieve a clinical pregnancy after transfer of at least 4 good-quality embryos in a minimum of three fresh or frozen cycles in a woman under the age of 40 years [6]. However, some clinicians have indicated that couples with two consecutive unsuccessful attempts of IVF-ET should also be included in RIF, similarly to the recent redefinition of recurrent pregnancy loss by the American Society for Reproductive Medicine (2013) [25]. In our study indications for IVF-ET were as follows: male factor (22.14%), female factor (22.14%), both factors (16.79%), or unexplained infertility (38.93%). The male factor consisted of abnormalities of semen in sperm count, vitality and motility and their morphology. The female factor comprised anatomic factors, including congenital uterine abnormalities, endometrial polyps, uterine fibroids, adhesions, hydrosalpinges, endometriosis, tubal factor, polycystic ovarian syndrome, ovulation dysfunction.

Homocysteine level was tested by immunoassay technique using the Immulite 2000 XPi analyzer.

The control group was selected in the 1st Department of Obstetrics and Gynecology, Medical University of Warsaw and the District Hospital Strzelce Opolskie. This group comprised 219 healthy couples with at least 2 healthy-born children and no history of miscarriage or endocrinological or immunological disorders. Details concerning this control group were described by Nowak et al. [26,27]. In addition, we decided to include 100 couples with one healthy-born child from anonymous paternity testing to increase the group of fertile couples to 319 to make our study stronger and more reliable. We also included their 100 children to assess the inheritance of *MTHFR* alleles. However, we did not possess any data about this group of fertile families, as they were anonymous participants in paternity cases. Detailed information about tested individuals is presented in Table 1. The study was approved by the Scientific Research Ethics Committee at the Polish Mothers’ Memorial Hospital Research Institute, no. 16/2014, and by the Local Ethics Committee of the Medical University of Wrocław. Informed written consent was obtained from the subjects.

**Table 1:** Characteristics of study population.

Attribute	Control	RSA	RIF
Maternal	N = 219	N = 289	N = 131
Age, mean ± SD; range	32.29 ± 5.81; 22–62	32.82 ± 4.02; 24–46	34.20 ± 3.61; 26–40
Paternal	N = 219	N = 282	N = 126
Age, mean ± SD; range	33.97 ± 6.18; 25–70	34.14 ± 3.19; 27–41	35.11 ± 4.34; 25–46
Indications for IVF-embryo transfer (%)	NA	NA	Male factor—29 (22.14)
			Female factor—29 (22.14)
			Both factors—22 (16.79)
			Unexplained infertility—51 (38.93)
Number of miscarriages after natural conception, mean ± SD; range	0	3.09 ± 1.24; 2–8	0
Number of pregnancies after IVF-ET, mean ± SD; range	NA	NA	1.08 ± 1.19; 0–5
Number of miscarriages after IVF-ET, mean ± SD; range	NA	NA	0.20 ± 0.40; 0–1
Number of previous implantation failures, mean ± SD; range	NA	NA	3.83 ± 1.54; 2–8
Number of embryo transfers, mean ± SD; range	NA	NA	4.66 ± 2.09; 2–12

RSA, recurrent spontaneous abortion; RIF, recurrent implantation failure; IVF-ET, *in vitro* fertilization–embryo transfer; NA, not applicable; SD, standard deviation; Male factor–abnormalities of semen in sperm count, vitality and motility; Female factor—endometriosis, tubal factor, polycystic ovarian syndrome, ovulation dysfunction; Data for the control group concern only 219 couples with 2 healthy-born children with the same partner. The remaining 100 couples from paternity testing were fully anonymous.

### DNA preparation and genotyping

Genomic DNA was isolated from venous blood by using the Invisorb Spin Blood Midi Kit (Invitek, Berlin, Germany) according to the manufacturer’s instructions. Procedures of *MTHFR* 677 C>T and 1298 A>C genotyping have been published recently in detail in supplementary material of our article [27].

### Statistical analysis

The frequencies of tested genotypes and alleles were estimated by direct counting. The genotype distributions of each single-nucleotide polymorphism in the case and control groups were compared by two-tailed Fisher’s exact test using GraphPad InStat version 3.06 software (San Diego, CA, USA). *p* < 0.05 was considered statistically significant. If *p* < 0.05, it was corrected (*p*_*corr*._) by the number of comparisons using Bonferroni correction. For 2x2 tables the odds ratio (OR) and 95% confidence interval for it were also calculated. Hardy-Weinberg equilibrium was checked using the chi-square test with one degree of freedom. All genotype frequencies were in Hardy-Weinberg equilibrium. We used FAMHAP version 19 software (http://famhap.meb.uni-bonn.de/) for estimation of haplotype frequencies and SHEsis software for calculation of differences between the control group and RSA and RIF groups [28].

## Results

To examine the association of *MTHFR* gene polymorphism with RSA and RIF, two single nucleotide polymorphisms, 677 C>T and 1298 A>C, were chosen. The genotype and minor allele frequencies for women and their partners from tested groups and controls are presented in S1 Table. We did not observe any differences in the frequencies of genotypes and minor allele frequency of the *MTHFR* 677 C>T between RSA or RIF women and fertile controls, or between their male partners and controls. In contrast, we found a protective effect of the 1298 AC genotype (*p* = 0.01, OR = 0.64) and carriers of the C allele (AC+CC genotypes; *p* = 0.009, OR = 0.65) against RSA, when comparing RSA and control women. No such effect was observed for RIF.

The combined 1298/677 genotype analysis is presented in S2 Table. AA/CT women were more frequent in RSA (31.14%) and RIF (25.20%) groups in comparison to fertile women (22.88%), but only in the case of RSA was this difference significant (*p* = 0.022, OR = 1.52), and after Bonferroni correction this significance was lost (*p*_*corr*_. = 0.15). Male combined genotype analysis revealed no association with reproductive failure of their female partners. What is more, no individuals were found homozygous for both mutations.

When we analyzed combined female/male genotype, we observed that AA/AC genotype of the 1298 *MTHFR* polymorphism was more frequent in RSA and RIF groups than in the control group, but only in the RSA group was borderline significance achieved (S3 Table, *p* = 0.049, OR = 1.49), which was lost after the correction for multiple testing (*p*_*corr*._ = 0.44). Furthermore, no association of female/male combined 677 C>T *MTHFR* genotypes was found with RSA and RIF.

Haplotype analysis is depicted in S4 Table. The frequency of haplotypes in women from all tested groups were similar except for C/C haplotype (1298C/677C). Significantly higher frequency of this haplotype was observed in the control group than the RSA group (*p* = 0.03, OR = 0.77). Also, the distribution of haplotypes in men did not indicate any association between male haplotypes and RSA or RIF.

Table 2 summarizes the effects of the *MTHFR* 1298 A>C and 677 C>T polymorphisms on the susceptibility to RSA.

**Table 2:** Summary of the effects of *MTHFR* 1298 A>C and 677 C>T polymorphisms on susceptibility to RSA.

Polymorphism	Associated genotype or haplotype	Comparison	Table	P, OR (95% CI)	Effect
Female 1298 A>C	AC	RSA vs Control	S1	0.010, 0.64 (0.45–0.89)	↓
Female 1298 A>C	AC+CC	RSA vs Control	S1	0.009, 0.65 (0.47–0.90)	↓
Female 1298 C>A/female 677 C>T	AA/CT	RSA vs Control	S2	0.022, 1.52 (1.07–2.19)	↑
Female 1298 A>C /male 1298 A>C	AA/AC	RSA couples vs Control couples	S3	0.049, 1.49 (1.01–2.17)	↑
Female 1298 A>C/677 C>T	C/C	RSA vs Control	S4	0.030, 0.77 (0.60–0.98)	↓

↓ protection; ↑ susceptibility

Because *MTHFR* polymorphism in positions 1298 and 677 could affect the level of homocysteine in body fluids, and, what is also important, we had available data for the presence or absence of homocysteine in plasma of the 249 RSA and 80 RIF women, we decided to include this clinical information in the statistical analysis. Only 19 (7.63%) patients from the RSA group and 13 (16.25%) from the RIF group possessed an elevated level of homocysteine. Table 3 illustrates the distribution of genotypes of tested polymorphisms in RSA and RIF groups according to the presence (≥12 μM/L) or absence (lack or below 12 μM/L) of homocysteine (Hcy). We did not find significant differences in distribution of the 1298 and 677 *MTHFR* genotypes between Hcy-positive and Hcy-negative patients.

**Table 3:** Distribution of genotypes in RSA and RIF groups according to the presence or absence of homocysteine (Hcy).

	**RSA**				**RIF**			
**1298 A>C**	**AA**	**AC**	**CC**	∑	**AA**	**AC**	**CC**	∑
**Hcy positive**	9	8	2	19	6	6	1	13
%	*47*.*37*	*42*.*10*	*10*.*53*	*100*	*46*.*15*	*46*.*15*	*7*.*70*	*100*
**Hcy negative**	121	88	21	230	38	24	5	67
%	*52*.*61*	*38*.*26*	*9*.*13*	*100*	*56*.*72*	*35*.*82*	*7*.*46*	*100*
**OR**	1	1.17	1.17	-	1	1.58	1.27	-
**CI 95%**	-	0.45–3.03	0.25–5.42	-	-	0.46–5.48	0.12–12.81	-
	χ^2^_df = 1_ = 0.18; *p* = 0.67				χ^2^_df = 1_ = 0.32; *p* = 0.57			
	**RSA**				**RIF**			
**677 C>T**	**CC**	**CT**	**TT**	∑	**CC**	**CT**	**TT**	∑
**Hcy positive**	6	9	4	19	6	5	2	13
%	*31*.*58*	*47*.*37*	*21*.*05*	*100*	*46*.*15*	*38*.*46*	*15*.*39*	*100*
**Hcy negative**	93	118	19	230	36	29	2	67
%	*40*.*44*	*51*.*30*	*8*.*26*	*100*	*53*.*73*	*43*.*28*	*2*.*99*	*100*
**OR**	1	0.85	2.96	-	1	1.03	6.00	-
**CI 95%**	-	0.34–2.18	0.89–9.82	-	-	0.29–3.73	0.70–51.13	-
	χ^2^_df = 1_ = 2.07; *p* = 0.15				χ^2^_df = 1_ = 1.24; *p* = 0.26			

RSA, recurrent spontaneous abortion; RIF, recurrent implantation failure; IVF-ET, *in vitro* fertilization–embryo transfer; P, probability; OR, odds ratio; 95% CI, 95% confidence interval from two-sided Fisher’s exact test; χ^2^_df = 1_, *p*, chi-square test with one degree of freedom.

## Discussion

In this study we aimed to investigate whether genetic *MTHFR* polymorphisms in 1298 A>C and 677 C>T are associated with reproductive failure, namely RSA and RIF. Moreover, we tried to elucidate the effect of *MTHFR* polymorphism on the homocysteine level in plasma of RSA and RIF patients. The main result is the protective effect of the *MTHFR* 1298 AC genotype (OR = 0.67, *p* = 0.01) and carriage of the C allele (AC+CC genotypes, OR = 0.65, *p* = 0.009) against RSA (S1 Table). The AC genotype frequency in our fertile female population was 47.65%, and it was similar to the frequency of 47.88% reported by Wolski et al. [29] from a study of 662 Polish women. Moreover, in our study, the frequency of the AC genotype of fertile women did not differ significantly from the frequency observed in fertile men. Also, the frequencies of combined 1298/677 genotypes were similar to those reported by Wolski et al. [29]. It should be noted that the protective 1298 AC genotype appeared mostly in combination with wild-type CC genotype of the 677 polymorphism, and it was more prevalent in the control group, although not significantly (S2 Table, *p* = 0.07). Furthermore, analysis of haplotypes proved that the 1298C allele protects from RSA only in the haplotype with the C allele in the 677 position (S4 Table). Therefore, we should test not only 1298 A>C *MTHFR* polymorphism but also 677 C>T, and each significant result should be considered with caution, because in our population the 1298 AC genotype with one mutated allele seems harmless. This mutation does not affect the thermolability or FAD release activity of the MTHFR enzyme [30,31]; however, mutation in 677 C>T could change the enzyme structure and the mRNA properties, as was reported recently by Nikzad et al. [32]. An *in silico* analysis of the wild-type and mutant 677 C>T mRNA indicated the removal of two loops in the secondary structure of mutant mRNA. Therefore, 222Val was more unstable than 222Ala in the MTHFR mRNA structure, which could influence expression of the protein [32].

Because we observed significantly higher frequency of the female 1298 AA/677 CT genotype in the RSA group, we might suppose that it could be important in susceptibility to RSA. However, the 1.52 odds ratio indicates a moderate risk of RSA for the 1298 AA/677 CT women (S2 Table). On the other hand, none of the combined genotypes of their partners showed a significant association. This result is consistent with a recently published meta-analysis by Pereza et al. [15], and a study on the German population, in which paternal thrombophilic gene mutations were not associated with RSA [33]. However, another study, which was reported by Tara et al. [34], is divergent with that outcome. Their results showed male heterozygosity in 1298 A>C/677 C>T polymorphisms as a predisposing factor to RSA of their partners. Moreover, *MTHFR*-Ala222Val (677 C>T) polymorphism has been recently associated with Iranian male infertility [32,35]. Indeed, scientists found the 222Val allele (677T) and Val-Val (677TT) genotype significantly more frequent in oligozoospermic and azoospermic men. However, in our population of men with severe and very severe oligozoospermia we did not find similar results (unpublished data), which is consistent with the research published by Wu et al. [36]. They reported a meta-analysis study stratified by ethnicity, which indicated a strong association between *MTHFR* 677 C>T polymorphism and male infertility only in Asians, but not with Caucasian men.

We also found no association of *MTHFR* polymorphisms with recurrent implantation failure, and this is in accordance with Di Nisio et al. [8], who in a review and meta-analysis found no association of female *MTHFR* mutations with reproductive failure after IVF. However, the number of RIF patients (N = 131) in our study seems to be insufficient. This is one of the limitation of our study. Possibly, increasing this group size would produce similar results as those we observed for miscarriages.

The combined female/male genotype analysis indicated a very weak association of only one AA/AC 1298 genotype with RSA, and it does not seem plausible, because the correction for multiple testing was *p*>0.05. In this case, 50% of fetuses would be AA (not mutated, with full enzyme activity) and 50% AC (with one mutated allele with minimal influence on the enzyme activity). On the other hand, analysis of maternal and fetal 1298 polymorphism in spontaneously aborted cases of the Indian population, and meta-analysis of 4 other studies, showed overrepresentation of the mutant alleles AC and CC [16]. However, the Indian population differs from the Caucasian population in many genetic aspects, and therefore our results could be different from the Asian population. Unfortunately, we had no available data concerning *MTHFR* polymorphisms from the fetal RSA group. The only information we had came from genotyping of 100 fertile families, and we may conclude that the inheritance of *MTHFR* follows a Mendelian pattern. Children did not differ from their mothers and fathers in genotype or haplotype frequencies (S5 Table). It should also be emphasized that no combined 1298 CC/677 TT homozygosity was observed in all our tested individuals. We cannot exclude that inheritance of such a genotype by an embryo could be lethal in very early embryo development. This observation has been reported in several studies [18,34].

An elevated female level of Hcy has been reported to impair DNA methylation and gene expression, causing defective chorionic villous vascularization in spontaneous miscarriage tissues [37]. Therefore, we included Hcy data in the analysis of a potential effect of *MTHFR* polymorphisms on the level of Hcy in plasma of RSA and RIF women. Analysis of the Hcy-positive and Hcy-negative RSA and RIF female patients with *MTHFR* genotypes revealed no effect of *MTHFR* polymorphism on the Hcy level. However, TT carriers of the 677 polymorphism in RSA and RIF more frequently possessed elevated levels of homocysteine, at increased odds ratios of 2.96 and 6.00, respectively. This is compatible with the study of Unfried et al. [38] showing that TT homozygosity in the *MTHFR* gene with hyperhomocysteinemia leads to a 3-fold increase in risk for early miscarriages. Moreover, Hekmatdoost et al. [17] found that plasma from idiopathic RSA patients with 1298 AC/677 TT genotype (three mutated alleles) differed significantly in Hcy value from plasma of patients with 1298 AA/677 CC genotype (non-mutated allele). Unfortunately, we were unable to make such an analysis because of the low numbers of Hcy-positive RSA and RIF patients, which is the second limitation of our study. Also, the lack of data on plasma Hcy concentrations in men as well as on the dietary folate intake in all tested individuals is a further limitation preventing us from drawing comprehensive conclusions from the study.

There are many reports of *MTHFR* polymorphisms with reproductive disorders, but they are unfortunately contradictory, so the conclusions from the meta-analyses are unclear. It might be a consequence of poor quality of the case-control studies, as has often been admitted by the authors [8,15,21]. Some studies showed no association between *MTHFR* polymorphisms and IVF outcomes and RSA, and do not recommend routine testing or treatment of thrombophilia. They proposed rather a careful personal estimation of thrombotic complication risk, especially for women after ovarian stimulation [9,39]. The prevalence of *MTHFR* 1298 and 677 mutations is high in the general population, so the anticoagulation treatment should not be indicated and only supplementation with folic acid is warranted. Indeed, in the clinical practice in Poland, we observed RSA patients with elevated Hcy which, after supplementation of folic acid, decreased to the reference level (unpublished observation). Therefore, we agree with others [9,39] that, for clinical practice, it is better to check the homocysteine level in plasma and, if the Hcy level is increased, to order patients to supplement themselves with folic acid rather than to undergo screening for *MTHFR* 1298 A>C and 677 C>T polymorphisms.

## Supporting information

S1 Table Genotype frequencies and minor allele frequencies of *MTHFR* 677 C>T and *MTHFR* 1298 A>C in controls, RSA, and RIF patients. (DOCX)

S2 Table Combined genotype frequencies of *MTHFR* 677 C>T and *MTHFR* 1298 A>C in controls, RSA, and RIF patients. (DOCX)

S3 Table Combined female/male genotype frequencies of *MTHFR* polymorphisms in controls, RSA, and RIF patients. (DOCX)

S4 Table Haplotype frequencies of the 1298 A>C and 677 C>T *MTHFR* polymorphisms in women and men from control, RSA and RIF groups. (DOCX)

S5 Table Genotype and haplotype frequencies of the 1298 A>C and 677 C>T *MTHFR* polymorphisms in 100 healthy families. (DOC)
