# Supplementary material for: The methylenetetrahydrofolate reductase c.c.677 C>T and c.c.1298 A>C polymorphisms in reproductive failures: Experience from an RSA and RIF study on a Polish population
Source: PLoS One. 2017 Oct 26;12(10):e0186022. doi: 10.1371/journal.pone.0186022 (PMC5657620; doi:10.1371/journal.pone.0186022)
Supplement: S1 Table — (DOCX) [file pone.0186022.s001.docx]

**S1 Table.** **Genotype frequencies and minor allele frequencies of *MTHFR* 677 C>T and *MTHFR* 1298 A>C in controls, RSA, and RIF patients.**

| **Genotype** | **Control (%)** | **RSA (%)** | **RIF (%)** | **RSA vs Control** | | | **RIF vs** **Control** | | |
| --- | --- | --- | --- | --- | --- | --- | --- | --- | --- |
|  |  |  |  | **P** | **OR (95% CI)** | **χ^2^_df=2_, *p*** | **P** | **OR (95% CI)** | **χ^2^_df=2_, *p*** |
| **677 C>T** |  |  |  |  |  |  |  |  |  |
| Female | N=319 | N=289 | N=131 |  |  |  |  |  |  |
| CC^a^ | 150 (47.02) | 120 (41.52) | 61 (46.56) |  | 1 | 1.88, 0.39 |  | 1 | 0.53, 0.77 |
| CT | 141 (44.20) | 142 (49.13) | 61 (46.56) | 0.20 | 1.26 (0.90-1.76) |  | 0.83 | 1.06 (0.70-1.62) |  |
| TT | 28 (8.78) | 27 (9.35) | 9 (6.87) | 0.55 | 1.21 (0.67-2.15) |  | 0.69 | 0.79 (0.35-1.77) |  |
| CT+TT | 169 (52.98) | 169 (58.48) | 70 (53.43) | 0.19 | 1.25 (0.91-1.72) |  | 1.00 | 1.02 (0.68-1.53) |  |
| Minor allele T | 197 (30.88) | 196 (33.91) | 79 (30.15) |  |  |  |  |  |  |
| H-W | 0.526 | 0.102 | 0.227 |  |  |  |  |  |  |
| Male | N=319 | N=282 | N=126 |  |  |  |  |  |  |
| CC^a^ | 156 (48.90) | 150 (53.19) | 59 (46.82) |  | 1 | 2.43, 0.30 |  | 1 | 1.38, 0.50 |
| CT | 134 (42.00) | 115 (40.78) | 59 (46.82) | 0.55 | 0.89 (0.64-1.25) |  | 0.51 | 1.16 (0.76-1.79) |  |
| TT | 29 (9.10) | 17 (6.03) | 8 (6.35) | 0.15 | 0.61 (0.32-1.16) |  | 0.55 | 0.73 (0.31-1.69) |  |
| CT+TT | 163 (51.10) | 132 (46.81) | 67 (53.17) | 0.33 | 0.84 (0.61-1.16) |  | 0.75 | 1.09 (0.72-1.64) |  |
| Minor allele T | 192 (30.09) | 149 (26.42) | 75 (29.76) |  |  |  |  |  |  |
| H-W | 0.977 | 0.411 | 0.178 |  |  |  |  |  |  |
| **1298 A>C** |  |  |  |  |  |  |  |  |  |
| Female | N=319 | N=289 | N=131 |  |  |  |  |  |  |
| AA^a^ | 133 (41.69) | 151 (52.25) | 63 (48.09) |  | 1 | **6.99, 0.03** |  | 1 | 1.57, 0.46 |
| AC | 152 (47.65) | 110 (38.06) | 55 (41.98) | **0.01** | **0.64 (0.45-0.89)** |  | 0.23 | 0.76 (0.50-1.17) |  |
| CC | 34 (10.66) | 28 (9.69) | 13 (9.92) | 0.27 | 0.73 (0.42-1.26) |  | 0.60 | 0.81 (0.40-1.63) |  |
| AC+CC | 186 (58.31) | 138 (47.75) | 68 (51.91) | **0.009** | **0.65 (0.47-0.90)** |  | 0.25 | 0.77 (0.51-1.16) |  |
| Minor allele C | 220 (34.48) | 166 (28.72) | 81 (30.92) |  |  |  |  |  |  |
| H-W | 0.330 | 0.232 | 0.845 |  |  |  |  |  |  |
| Male | N=319 | N=282 | N=126 |  |  |  |  |  |  |
| AA^a^ | 146 (45.77) | 123 (43.62) | 57 (45.24) |  | 1 | 0.34, 0.84 |  | 1 | 0.30, 0.86 |
| AC | 143 (44.83) | 133 (47.16) | 55 (43.65) | 0.61 | 1.10 (0.79-1.55) |  | 1.00 | 0.98 (0.64-1.52) |  |
| CC | 30 (9.40) | 26 (9.22) | 14 (11.11) | 1.00 | 1.03 (0.58-1.83) |  | 0.71 | 1.19 (0.59-2.42) |  |
| AC+CC | 173 (54.23) | 159 (56.38) | 69 (54.76) | 0.60 | 1.09 (0.79-1.51) |  | 1.00 | 1.02 (0.67-1.55) |  |
| Minor allele C | 203 (31.82) | 185 (32.80) | 83 (32.94) |  |  |  |  |  |  |
| H-W | 0.554 | 0.241 | 0.894 |  |  |  |  |  |  |

RSA, recurrent spontaneous abortion; RIF, recurrent implantation failure; H-W, Hardy-Weinberg equilibrium; P, probability; OR, odds ratio; 95% CI, 95% confidence interval from two-sided Fisher’s exact test; χ^2^_df=2_, *p* chi-square test with two degree of freedom; ^a^Reference
