# Supplementary material for: The methylenetetrahydrofolate reductase c.c.677 C>T and c.c.1298 A>C polymorphisms in reproductive failures: Experience from an RSA and RIF study on a Polish population
Source: PLoS One. 2017 Oct 26;12(10):e0186022. doi: 10.1371/journal.pone.0186022 (PMC5657620; doi:10.1371/journal.pone.0186022)
Supplement: S2 Table — (DOCX) [file pone.0186022.s002.docx]

**S2 Table.** **Combined genotype frequencies of *MTHFR* 677 C>T and *MTHFR* 1298 A>C in controls, RSA, and RIF patients.**

| **Combined genotypes** | | **Control** | **RSA** | **RIF** | **RSA vs Control** | | **RIF vs** **Control** | |
| --- | --- | --- | --- | --- | --- | --- | --- | --- |
|  |  |  |  |  | **P** | **OR (95% CI)** | **P** | **OR (95% CI)** |
| **1298 A>C** | **677 C>T** |  |  |  |  |  |  |  |
| Female |  | N=319 | N=289 | N=131 |  |  |  |  |
| AA | CC | 32 (10.03) | 34 (11.76) | 21 (16.03) | 0.52 | 1.20 (0.71-2.02) | 0.08 | 1.71 (0.95-3.10) |
| AA | CT | 73 (22.88) | 90 (31.14) | 33 (25.20) | **0.022^a^** | **1.52 (1.07-2.19)** | 0.63 | 1.13 (0.71-1.82) |
| AA | TT | 28 (8.78) | 27 (9.34) | 9 (6.87) | 0.89 | 1.07 (0.63-1.90) | 0.57 | 0.77 (0.35-1.67) |
| AC | CC | 86 (26.96) | 59 (20.42) | 27 (20.61) | 0.07 | 0.70 (0.47-1.02) | 0.19 | 0.70 (0.43-1.15) |
| AC | CT | 66 (20.69) | 51 (17.65) | 28 (21.37) | 0.36 | 0.82 (0.55-1.81) | 0.90 | 1.04 (0.63-1.71) |
| AC | TT | 0 (0.0) | 0 (0.0) | 0 (0.0) | - | - | - | - |
| CC | CC | 32 (10.03) | 27 (9.34) | 13 (9.92) | 0.79 | 0.92 (0.53-1.57) | 1.00 | 0.99 (0.50-1.95) |
| CC | CT | 2 (0.63) | 1 (0.35) | 0 (0.0) | 1.00 | 0.55 (0.04-4.76) | 1.00 | 0.48 (0.02-10.13) |
| CC | TT | 0 (0.0) | 0 (0.0) | 0 (0.0) | - | - | - | - |
| Male |  | N=319 | N=282 | N=126 |  |  |  |  |
| AA | CC | 56 (17.56) | 46 (16.31) | 18 (14.29) | 0.74 | 0.92 (0.60-1.40) | 0.48 | 0.78 (0.44-1.39) |
| AA | CT | 61 (19.12) | 60 (21.28) | 31 (24.60) | 0.54 | 1.14 (0.77-1.69) | 0.20 | 1.38 (0.84-2.26) |
| AA | TT | 29 (9.10) | 17 (6.03) | 8 (6.35) | 0.17 | 0.64 (0.35-1.20) | 0.45 | 0.68 (0.30-1.53) |
| AC | CC | 70 (21.94) | 78 (27.66) | 27 (21.43) | 0.11 | 1.36 (0.93-1.96) | 1.00 | 0.97 (0.59-1.60) |
| AC | CT | 73 (22.88) | 55 (19.50) | 28 (22.22) | 0.32 | 0.82 (0.55-1.22) | 1.00 | 0.96 (0.59-1.58) |
| AC | TT | 0 (0.0) | 0 (0.0) | 0 (0.0) | - | - | - | - |
| CC | CC | 30 (9.40) | 26 (9.22) | 14 (11.11) | 1.00 | 0.98 (0.57-1.71) | 0.60 | 1.20 (0.62-2.36) |
| CC | CT | 0 (0.0) | 0 (0.0) | 0 (0.0) | - | - | - | - |
| CC | TT | 0 (0.0) | 0 (0.0) | 0 (0.0) | - | - | - | - |

RSA, recurrent spontaneous abortion; RIF, recurrent implantation failure; P, probability; OR, odds ratio; 95% CI, 95% confidence interval from two-sided Fisher’s exact test; ^a^*p_corr._*= 0.15
