# Supplementary material for: The methylenetetrahydrofolate reductase c.c.677 C>T and c.c.1298 A>C polymorphisms in reproductive failures: Experience from an RSA and RIF study on a Polish population
Source: PLoS One. 2017 Oct 26;12(10):e0186022. doi: 10.1371/journal.pone.0186022 (PMC5657620; doi:10.1371/journal.pone.0186022)
Supplement: S3 Table — (DOCX) [file pone.0186022.s003.docx]

| **Female/male combined genotype** | | **Control (%)** | **RSA (%)** | **RIF (%)** | **RSA vs Control** | | **RIF vs** **Control** | |
| --- | --- | --- | --- | --- | --- | --- | --- | --- |
|  |  |  |  |  | **P** | **OR (95% CI)** | **P** | **OR (95% CI)** |
| **1298 A>C** | | N=319 | N=282 | N=126 |  |  |  |  |
| AA | AA | 67 (21.00) | 64 (22.70) | 27 (21.43) | 0.77 | 1.07 (0.72-1.58) | 0.89 | 1.03 (0.62-1.70) |
| AA | AC | 59 (18.50) | 73 (25.89) | 31 (24.60) | **0.049^a^** | **1.49 (1.01-2.17)** | 0.15 | 1.44 (0.88-2.36) |
| AA | CC | 7 (2.19) | 13 (4.61) | 4 (3.17) | 0.11 | 2.15 (0.83-5.61) | 0.51 | 1.46 (0.42-5.08) |
| AC | AA | 66 (20.69) | 48 (17.02) | 26 (20.63) | 0.30 | 0.79 (0.52-1.19) | 1.00 | 1.00 (0.60-1.66) |
| AC | AC | 65 (20.38) | 46 (16.31) | 17 (13.49) | 0.21 | 0.76 (0.50-1.16) | 0.10 | 0.61 (0.34-1.09) |
| AC | CC | 21 (6.58) | 10 (3.55) | 8 (6.35) | 0.35 | 0.67 (0.32-1.47) | 1.00 | 0.96 (0.41-2.23) |
| CC | AA | 13 (4.08) | 11 (3.90) | 4 (3.17) | 1.00 | 0.96 (0.43-2.14) | 0.79 | 0.77 (0.25-2.41) |
| CC | AC | 19 (5.96) | 14 (4.96) | 7 (5.56) | 0.72 | 0.82 (0.40-1.69) | 1.00 | 0.93 (0.38-2.27) |
| CC | CC | 2 (0.62) | 3 (1.06) | 2 (1.60) | 0.67 | 1.70 (0.35-9.65) | 0.32 | 2.56 (0.36-18.36) |
| **677 C>T** | | N=319 | N=282 | N=126 |  |  |  |  |
| CC | CC | 78 (24.45) | 58 (20.57) | 24 (19.06) | 0.28 | 0.78 (0.54-1.18) | 0.26 | 0.73 (0.44-1.21) |
| CC | CT | 62 (19.44) | 50 (17.73) | 30 (23.81) | 0.60 | 0.89 (0.59-1.35) | 0.30 | 1.29 (0.79-2.12) |
| CC | TT | 10 (3.13) | 8 (2.84) | 4 (3.17) | 1.00 | 0.90 (0.35-2.32) | 1.00 | 1.01 (0.31-3.29) |
| CT | CC | 66 (20.70) | 75 (26.59) | 31 (24.60) | 0.10 | 1.39 (0.95-2.03) | 0.37 | 1.25 (0.77-2.04) |
| CT | CT | 60 (18.81) | 56 (19.86) | 25 (19.85) | 0.76 | 1.07 (0.71-1.61) | 0.79 | 1.07 (0.64-1.80) |
| CT | TT | 15 (4.70) | 9 (3.19) | 4 (3.17) | 0.41 | 0.67 (0.29-1.55) | 0.61 | 0.66 (0.22-2.04) |
| TT | CC | 12 (3.76) | 17 (6.03) | 4 (3.17) | 0.25 | 1.64 (0.77-3.50) | 1.00 | 0.84 (0.27-2.65) |
| TT | CT | 12 (3.76) | 9 (3.19) | 4 (3.17) | 0.83 | 0.84 (0.35-2.03) | 1.00 | 0.84 (0.27-2.65) |
| TT | TT | 4 (1.25) | 0 (0.00) | 0 (0.00) | 0.13 | 0.12 (0.01-2.32) | 0.58 | 0.28 (0.01-5.19) |

**S3 Table. Combined female/male genotype frequencies of *MTHFR* polymorphisms in controls, RSA, and RIF patients.**

RSA, recurrent spontaneous abortion; RIF, recurrent implantation failure; P, probability; OR, odds ratio; 95% CI, 95% confidence interval from two-sided Fisher’s exact test; *^a^p_corr._*= 0.44
