# Supplementary material for: The methylenetetrahydrofolate reductase c.c.677 C>T and c.c.1298 A>C polymorphisms in reproductive failures: Experience from an RSA and RIF study on a Polish population
Source: PLoS One. 2017 Oct 26;12(10):e0186022. doi: 10.1371/journal.pone.0186022 (PMC5657620; doi:10.1371/journal.pone.0186022)
Supplement: S4 Table — (DOCX) [file pone.0186022.s004.docx]

**S4 Table. Haplotype frequencies of the 1298 A>C and 677 C>T *MTHFR* polymorphisms in women and men from control, RSA and RIF groups.**

| **Haplotype 1298/677** | **Control %** | **RSA %** | **RIF %** | **RSA vs Control** | | **RIF vs** **Control** | | **Global χ^2^_df=2_, *p*** | |
| --- | --- | --- | --- | --- | --- | --- | --- | --- | --- |
|  |  |  |  | **P** | **OR (95% CI)** | **P** | **OR (95% CI)** | **RSA vs Control** | **RIF vs** **Control** |
| **Female** | N=319 | N=289 | N=131 |  |  |  |  |  |  |
| A/C | 35.10 | 37.60 | 38.93 | 0.38 | 1.11 (0.88-1.41) | 0.28 | 1.18 (0.87-1.58) | 4.50, 0.11 | 1.28, 0.53 |
| A/T | 30.40 | 33.60 | 30.15 | 0.23 | 1.16 (0.91-1.47) | 1.00 | 0.99 (0.72-1.35) |  |  |
| C/C | 34.00 | 28.50 | 30.92 | **0.03** | **0.77 (0.60-0.98)** | 0.39 | 0.87 (0.64-1.18) |  |  |
| C/T | 0.50 | 0.30 | 0.00 | - | - | - | - | - | - |
| **Male** | N=319 | N=282 | N=126 |  |  |  |  |  |  |
| A/C | 38.10 | 40.80 | 37.30 | 0.34 | 1.12 (0.89-1.41) | 0.88 | 0.97 (0.71-1.31) | 2.07, 0.36 | 0.11, 0.95 |
| A/T | 30.10 | 26.40 | 29.76 | 0.16 | 0.83 (0.65-1.07) | 0.94 | 0.98 (0.71-1.35) |  |  |
| C/C | 31.80 | 32.80 | 32.94 | 0.72 | 1.05 (0.82-1.33) | 0.75 | 1.05 (0.77-1.43) |  |  |
| C/T | 0.00 | 0.00 | 0.00 | - | - | - | - | - | - |

RSA, recurrent spontaneous abortion; RIF, recurrent implantation failure; P, probability; OR, odds ratio; 95% CI, 95% confidence interval from two-sided Fisher’s exact test; χ^2^_df=2_, *p*, global chi-square test with two degree of freedom.
