# Supplementary material for: The methylenetetrahydrofolate reductase c.c.677 C>T and c.c.1298 A>C polymorphisms in reproductive failures: Experience from an RSA and RIF study on a Polish population
Source: PLoS One. 2017 Oct 26;12(10):e0186022. doi: 10.1371/journal.pone.0186022 (PMC5657620; doi:10.1371/journal.pone.0186022)
Supplement: S5 Table — (DOC) [file pone.0186022.s005.doc]

**S5 Table. Genotype and haplotype frequencies of the 1298 A>C and 677 C>T *MTHFR* polymorphisms in 100 healthy** families.

|  | **Females** | **Males** | **Children** | **Children vs Females** | | | **Children vs Males** | | | **Males vs Females** | | |
| --- | --- | --- | --- | --- | --- | --- | --- | --- | --- | --- | --- | --- |
|  |  |  |  | **P** | **OR (95% CI)** | **χ^2^, *p*** | **P** | **OR (95% CI)** | **χ^2^, *p*** | **P** | **OR (95% CI)** | **χ^2^, *p*** |
| **Genotype** | N=100 | N=100 | N=100 |  |  |  |  |  |  |  |  |  |
| AA^a^ | 42.00 | 38.00 | 40.00 |  | 1 | 0.19, 0.91 |  | 1 | 0.10, 0.95 |  | 1 | 0.37, 0.83 |
| AC | 45.00 | 49.00 | 48.00 | 0.78 | 1.13 (0.65-1.97) |  | 1.00 | 0.96 (0.55-1.67) |  | 0.67 | 1.17 (0.67-2.05) |  |
| CC | 13.00 | 13.00 | 12.00 | 1.00 | 0.91 (0.39-2.11) |  | 1.00 | 0.91 (0.39-2.11) |  | 1.00 | 1.17 (0.44-2.28) |  |
| H-W | 0.86 | 0.65 | 0.68 |  |  |  |  |  |  |  |  |  |
| CC^a^ | 47.00 | 51.00 | 46.00 |  | 1 | 0.07, 0.97 |  | 1 | 0.55, 0.76 |  | 1 | 0.52, 0.77 |
| CT | 45.00 | 40.00 | 45.00 | 1.00 | 1.11 (0.57-1.75) |  | 0.57 | 1.23 (0.70-2.15) |  | 0.57 | 0.81 (0.46-1.43) |  |
| TT | 8.00 | 9.00 | 9.00 | 1.00 | 1.14 (0.42-3.08) |  | 1.00 | 1.19 (0.38-2.64) |  | 1.00 | 1.14 (0.42-3.08) |  |
| H-W | 0.54 | 0.77 | 0.67 |  |  |  |  |  |  |  |  |  |
| **Haplotype** |  |  |  |  |  |  |  |  |  |  |  |  |
| A/C | 35.50 | 33.50 | 32.50 | 0.52 | 0.87 (0.58-1.32) | 3.63, 0.30 | 0.83 | 0.96 (0.63-1.45) | 0.30, 0.86 | 0.67 | 1.09 (0.72-1.65) | 3.50, 0.32 |
| A/T | 29.00 | 29.00 | 31.50 | 0.58 | 1.13 (0.74-1.73) |  | 0.59 | 1.13 (0.74-1.73) |  | 1.00 | 1.00 (0.65-1.54) |  |
| C/C | 34.00 | 37.50 | 36.00 | 0.67 | 1.09 (0.73-1.65) |  | 0.76 | 0.94 (0.62-1.41) |  | 0.46 | 0.86 (0.57-1.29) |  |
| C/T | 1.50 | 0.00 | 0.00 | 0.13 | 0.00 (0.00-0.02) |  | - | - |  | 0.13 | 1188.69 (61.55-22957) |  |

H-W, Hardy-Weinberg equilibrium; P, probability; OR, odds ratio; 95% CI, 95% confidence interval from two-sided Fisher’s exact test; χ^2^_df=2_, p chi-square test with two degree of freedom; ^a^Reference
